# Possibilities and Hindrances for Prevention of Intimate Partner Violence: Perceptions Among Professionals and Decision Makers in a Swedish Medium-Sized Town

**DOI:** 10.1007/s12529-012-9238-1

**Published:** 2012-05-25

**Authors:** A. Jakobsson, C. von Borgstede, G. Krantz, F. Spak, G. Hensing

**Affiliations:** 1 Department of Psychology, University of Gothenburg, Gothenburg, Sweden; 2 Department of Public Health and Community Medicine/Social Medicine, The Sahlgrenska Academy at University of Gothenburg, Box 453, 405 30 Gothenburg, Sweden

**Keywords:** Intimate partner violence, Domestic violence, Prevention, Shared responsibility, Qualitative method

## Abstract

**Background:**

Intimate partner violence (IPV) is a major public health problem, but few evidence-based prevention programs have yet been implemented.

**Purpose:**

This study explored the perceptions and beliefs of local-level decision makers, social and health-care professionals, and representatives from the police force regarding the possibilities and hindrances for prevention of IPV.

**Method:**

An explorative qualitative approach was used, and participants were strategically selected for focus group discussions. The participants, 19 men and 23 women, were professionals or decision makers within health-care services, social welfare, municipal administration, the police force, local industry, and local politicians in a Swedish town of 54,000 inhabitants. The focus group discussions were audio recorded, transcribed verbatim, and thematically analyzed. A manifest content analysis was performed on the text.

**Results:**

Preschools, schools, sports associations, workplaces, and the mass media were suggested as possible arenas for prevention measures. The proposed activities included norm building and improved social support structures. Hindrances were conceptualized as societal beliefs and attitudes, shame, silence, gender inequality, the counteracting influence of the media, and lack of resources. The participants demonstrated closeness and distance to IPV, expressed as acceptance or referral of responsibility to others regarding where and by whom prevention measures should be executed.

**Conclusion:**

This study gave new insights in the prevailing perceptions of professionals and decision makers of a medium-sized Swedish town, which can be a useful knowledge in future preventive work and contribute to bridge the gap between research and practice.

## Introduction

Intimate partner violence (IPV) is a major public health problem both throughout the world and in Sweden [1, 2]. However, despite high prevalence, extensive health consequences, and societal costs, few evidence-based preventive interventions have been implemented [3].

The American Centers for Disease Control and Prevention concluded that there is a need for a prevention approach that targets contexts such as communities, schools, legislative environments, and other policy arenas [4]. There already exist school projects which challenge students' attitudes, norms, and behaviors, but assessments so far have shown only small and short-lived effects of such interventions. A lower prevalence of violence is found in countries with legislation against IPV, which often coexists with a higher level of gender equity [3]. However, the phenomenon still persists even in countries with explicit legislation against IPV, which suggests that more arenas should be involved in preventive activities.

According to Coker [5], health-care providers have the responsibility to identify health threats such as IPV and provide relevant strategies to prevent the violence and its consequences. A systematic review by Casteel and Sadowski [6] showed that interventions initiated by health-care professionals aimed at women victims of IPV, such as advocacy, cognitive behavioral counseling, trauma therapy, peer support groups, and safety planning are likely to be beneficial. Other forms of counseling, shelters, and nurse support showed unknown effectiveness or were unlikely to be advantageous. Screening of women exposed to IPV and referral to local health and social services when needed has been recommended by medical organizations [7]. However, except for the lack of evidence of its effectiveness [8], a literature review by Rönnberg and Hammarström [9] revealed barriers which could stop health-care workers intervening against sexualized violence, such as lack of education, time constraints, stereotypical views of battered women, and concern over offending the victim or abuser.

Holmberg and Bender [10] reported in 2003 that in Sweden, both politicians and social workers were aware of the negative effects of IPV, but few measures were taken to prevent it or to protect women who are exposed to violence. Women's shelters were usually organized and run by nongovernmental organizations with little support from local authorities, and legislation stating the responsibilities of municipalities was lacking. One reason for this could be that IPV is regarded as a private matter rather than a political one, which impedes the issue from being discussed and acted on.

Hyman et al. [11] have suggested that the most appropriate and cost-effective method of preventing IPV would be proactive primary prevention, such as educational or policy-related interventions that actually lead to change. Researchers agree on the need for interventions on multiple levels in organizations and among professionals, if violence against women is to be prevented [1, 11]. It is thus important to be aware of how decision makers and other professionals view their possibilities to act, but investigations of this aspect are still lacking. This study was designed to explore the perceptions and beliefs of local-level decision makers, social and health-care professionals, local business people, and representatives from the police force regarding the possibilities and hindrances for the prevention of IPV. The intention was that the findings from the study could be used to develop strategies to influence the adoption of evidence-based interventions.

## Methods

### Study Design and Subject Recruitment

Focus group discussions were used for data collection, since this method is particularly well suited for exploring people's shared knowledge and experiences. Furthermore, interaction between the participants encourages clarification of their views [12], promotes a deeper understanding of the problem under study, and might give rise to new hypotheses [13].

### Study Participants

The focus group participants were strategically selected from predecided professional groups and decision makers within health-care services, social welfare, municipal administration, the police force, local industry, and local politicians. The purpose was to obtain as much variation as possible according to level of direct or indirect contact with IPV and experiences of prevention. The recruitment was performed in collaboration with a public health coordinator in a medium-sized Swedish town (54,000 inhabitants). The public health coordinator selected key persons who represented the predecided groups, and information about the study was then sent to them by mail, followed by telephone calls asking for their participation. The vast majority of the proposed focus group participants agreed to take part in the study. The few who declined participation did so because of time constraints.

Seven focus groups were formed of decision makers at different levels in the local community, representatives from local businesses, professionals, and authorities involved in public welfare and care (Table 1). Each focus group consisted of five to seven individuals [14]. Some of the groups were homogeneous in terms of profession, while others were heterogeneous, including participants of various professions. Five of the groups were mixed sex and two were purposely composed of exclusively men and women, to ensure that a broad variety of notions was captured. In all, 42 respondents agreed to participate, 23 women and 19 men. The dates and locations of the discussions were set according to the respondents' convenience. Five of the focus group discussions were carried out in an official room in the town hall, and the remaining two, at different workplaces.

**Table 1:** Characteristics of the focus groups

Group	Men/women (*n*)	Specialized area
I	1/5	Professionals—health care
II	0/6	Professionals—social welfare
III	5/0	Professionals—police force
IV	3/3	Decision makers—politics
V	2/4	Decision makers—local business and industrial organizations
VI	4/2	Decision makers—municipality management
VII	4/3	Decision makers—police authority

Each focus group discussion was led by a moderator and a comoderator. The moderator focused on the topic whereas the comoderator made observations, took notes, and asked complementary questions. The mixed groups were led by a female moderator and a male comoderator, the female focus group discussions (FGD) by two women, and the male FGD by two men.

### Procedures

The focus group discussions were semistructured and had an explorative character. After giving information about the project and introduction to the topic of the discussion, the following thematic questions were discussed: (1) what can you as professionals/decision makers do to prevent men's violence against women, (2) what are the possibilities for prevention of IPV in your community, and (3) what are the hindrances to implement prevention measures in your community? In order to obtain rich and comprehensive data and to stimulate the discussion, probes such as “can you explain some more” or “can you give an example” were used.

To test the thematic questions, a pilot investigation was conducted with faculty members from the Institute of Health and Caring Sciences at Goteborg University. No major changes were made after the test.

### Analysis

The focus group sessions were audio recorded and transcribed verbatim. The transcripts were analyzed by a moderator (CvB) in cooperation with a comoderator (AJ), using a qualitative analysis based on the manifest content of the text [15] and taking a phenomenological approach to interpretation of the participants' lived experiences [16].

After listening to the tapes several times, the transcripts were analyzed line by line in search for meaning units by CvB and AJ separately. All meaning units were then listed, condensed, and grouped according to content by CvB and AJ in collaboration. The grouping was discussed and disagreements were processed until an agreement was reached. Tentative categories based on the grouped content were identified and presented at a seminar where researchers and practitioners outside the project group discussed the findings. Finally, the authors sorted, conceptualized, and abstracted the categories which resulted in two main themes that captured the content of the data [13].

### Ethical Considerations

The design of the study was reviewed by the regional ethical committee, and no objections were raised. Informed consent was obtained from all participants before the focus group discussions started. The participants were informed about the study and assured that the data would be treated with confidentiality when published.

## Results

The findings were organized into three themes: *prevention proposals*, *hindrances*, *and closeness and distance to IPV* (see Table 2). A detailed presentation of the themes and the categories related to each theme is presented below.

**Table 2:** The themes and associated categories

Themes	Prevention proposals		Hindrances	Closeness and distance to IPV	
Categories	Norm building	Improving social support structures	Societal beliefs and lack of commitment	Acceptance or referral of responsibility	Professional disillusion

### Prevention Proposals

The focus group participants suggested that prevention should take place in preschools, schools, sports associations, workplaces, and mass media. In general, children and adolescents were considered to be the most suitable target groups for prevention strategies, since these groups were regarded as being more open to such interventions.

#### Norm Building

The suggested prevention activities ranged from educational programs directed towards specific target groups, to an everyday approach including creating and promoting conversations about IPV prevention. One suggestion was that domestic violence could be brought up in conversations between employers and employees in the workplace, and thus an anti IPV norm could be encouraged. Alcohol and drug abuse was regarded as a common cause of IPV, thus prevention of substance abuse in schools was considered an important IPV prevention strategy. The most important strategy for the prevention of IPV was considered to be political decision making contributing to change of societal norms regarding IPV.

The focus group participants suggested several places where people meet as suited for norm building activities. One example of a specific activity for adults was the creation of networks for men, aimed at providing arenas to discuss and problematize attitudes regarding violence against women. Another suggestion was to always show moral courage, stand up for one's beliefs, and authoritatively condemn IPV:

“… it is very important to show that there are definite norms here [on IPV^1^] either within this or that company or within this or that school, and that these norms are the only ones that can be accepted.” (*Private businessman*)

The participants proposed that norm building regarding IPV should start early in life, preferably at preschool:

“…just to start talking about it among friends and colleagues is a way to become aware, and I think it has to start with children, in school and pre-school…”

(*Social worker, woman*)

Educational programs in schools at various educational levels were regarded as important norm building activities. Suggested topics for these programs were conflict management, communication skills, developing adult relationships, and peaceful coexistence. The participants recommended that schools should use affirmative role models as a way to promote healthy relationships.

The media was regarded as an important arena for norm building. However, the participants believed that the role of media for norm building was complicated, since the media can also be used to create and establish undesirable norms. Specifically, limiting children's exposure to stereotyped media images of aggressive men and objectified women was suggested.

#### Improving Social Support Structures

Creating new social, political, and professional collaboration systems was regarded as an important step which would improve the handling and distribution of the prevention responsibilities. Improving the support systems for vulnerable families in health and social care was also recommended, as was increased financial aid to shelters providing support to victims of IPV. The participants believed that societal responsibilities also should include intentions to prevent an abuser from being violent in new relationships and provide support to children who are affected by IPV. Another suggestion was that screening for IPV could take place as a standard procedure by health-care providers addressing both women and men:

“…It could be preventive in a long term perspective if health-care centers started asking both men and women questions about IPV, because then men will know that these types of questions will be asked…”

(*Health-care provider, women*)

The need for increased resources to promote long-term prevention activities was repeatedly stressed. Collaboration between different professional organizations was seen as a way to create a more efficient handling of prevention of IPV. The participants also suggested that a clear division of responsibilities between different organizations could promote the prevention efforts.

### Hindrances

The participants identified several problems regarding the prevention of IPV. These problems were attributed to both community and individual level.

#### Societal Beliefs and Lack of Commitment

The focus group participants thought that the worst hindrances were shame, silence, gender inequality, the counteracting influence of the mass media, and lack of financial and professional resources. Shame was perceived as a reason why the question of IPV is neglected in society and why both victims and abusers are silent.

“…a man's violence against women is a shortcoming, so I think it is very shameful… it is shame enough to be beaten, but I think it is even more shameful to beat…”

(*Social worker, woman*)

Lack of knowledge and commitment regarding IPV among professionals and decision makers hampered the implementation of prevention strategies. Some of the participants emphasized the belief that the prevalence of IPV needs to be acknowledged and pointed to a huge number of unknown cases of IPV. Their opinion was that the hidden cases must be detected before prevention measures can be initiated. However, the police officers opposed this view and called attention to other situations when implementations of preventive strategies are not held back due to hidden cases:

“… we know that we only catch a small proportion of drunk drivers, but that doesn't stop us from setting up routine sobriety checkpoints, even though we only see the ‘tip of the iceberg’.”

(*Police officer, man*)

### Closeness and Distance to IPV

The participants' ideas of how and where prevention strategies could be effective were influenced by their lived experience, including their previous professional contacts with IPV. The theme “closeness and distance to IPV” emanated from these ideas and included two categories: “acceptance or referral of responsibility” and “professional disillusion”*.*

#### Acceptance or Referral of Responsibility

Professionals and decision makers shared the belief that IPV is an important public health problem and that there is a need for some authorities to accept responsibility, but their attitudes toward accepting this responsibility ranged from enthusiasm to despondency. Police officers and politicians had different points of departure concerning what should be done to prevent IPV and who should do it. The politicians realized their possibilities to contribute, whereas the police officers were more doubtful concerning their role in preventive work. Although the professionals realized that they could have a role to play in preventing IPV, they thought that another profession or organization might be more suited for the work. The politicians, on the other hand, accepted responsibility and were confident in their roles as leaders, feeling that they could create an open discussion as well as influence the public opinion, even if there was some resistance.

“…I think we have a lot to do, but it's our mutual responsibility now”

*(Politician, woman)*

#### Professional Disillusion

The professionals experienced IPV as a difficult and recurrent social problem and were concerned with the limitations they saw regarding possible preventive actions. They expressed frustration and lack of hope, particularly when they talked about insufficient support for the victims of violence. Both police officers and social workers supported women exposed to IPV in several ways, yet they had experienced several hindering factors which affected their ability to provide the prevention and support they wanted, including limited time, lack of financial resources, and other challenging duties. Professional disillusion was also created by insufficient evidence of a positive outcome when legal action was taken.

“…she called when there was trouble, so the fellow was arrested and we would go and hold a hearing in which she absolutely refused to participate”

(*Police officer, man)*

The professionals expressed frustration and hopelessness, due to engagement in many difficult problems in their daily work. They also expressed a lack of professional effectiveness, which provoked feelings of despair:

“…you work and work and try to help, and it all ends up in a disaster anyway*…*”

*(Police officer, man)*

Both police officers and social workers felt that their experiences and positions close to the victims of violence were advantageous, in particular when dealing with IPV on a secondary prevention level.

## Discussion

There were two main findings from this focus group study regarding prevention of IPV. The first concerned the proposals for interventions from the focus groups and these were (1) to improve social support structures through political decision making and (2) to promote norm-building actions, preferably in preschool, schools, workplaces, and through media. The second main finding was that participants stressed the urgent need for implementation of prevention strategies, but the professionals and the decision makers differed in terms of how they believed their own profession could contribute. The health-care providers, the social workers, and the police officers expressed reluctance to shoulder their responsibility of IPV prevention, whereas the decision makers considered themselves able to contribute in a fruitful way.

### Social Support Structures

Early detection of IPV by routine screening of both women and men was proposed by the participants in this study as was increased financial support for shelters and increased support for vulnerable families. The evidence for routine screening by health-care providers is still not sufficient for recommendations [8]; still, increased awareness and inquiring about exposure to IPV in patients who have signs or symptoms might contribute to early detection and be beneficial for some women [17]. Shelter stay on its own has unknown effectiveness [6] but might be beneficial in preventing reabuse in women if specific programs of advocacy or counseling was included [8]. Increased collaboration between political, social, and professional systems was also suggested in this study. Efforts have been made in the USA to evaluate primary and secondary prevention activities targeting community beliefs about IPV, increased victim assistance, and increased accountability for perpetrators. The evaluation showed that the prevention activities had little effect on the frequency of IPV. However, it showed that coordinated responses to IPV may increase contact with IPV services when goals are based on the salience of the need in the community [18].

### Changing Norms

Preschools and schools were suggested as arenas for IPV prevention, with the main intention to change norms and provide children and young people with useful alternative skills to resolve conflicts. However, so far, IPV prevention in schools has resulted in only limited behavioral changes [3]. Swedish and international assessments have also shown scarce effects of educational programs addressing other public health problems, e.g., alcohol prevention [19]. The idea of changing social norms and promote nonacceptance of IPV is in line with previous research [1, 3]. However, the challenge is to create efficient norm changing programs that actually lead to behavioral change. Norms are not given but need to be inferred, and this occurs in social interaction by communicating and observing the behavior of others [20]. The socialization of people into new norms involves not only what is said but also what is left unsaid [21]. Social norms evolve to regulate social life and serve to maintain a social order, particularly when the behaviors of individuals cause negative side effects for others [22]. The participants emphasized that norm building occurs in daily interpersonal social interactions and that everyone has a personal responsibility to dissociate from violence of any kind. This perception is supported by research showing that norms emerge in interactions in everyday life situations between friends or between colleagues at work, and these norms are known as general interaction norms [23].

The incongruence between the notions of more formal programs to change social norms suggested by the focus group participants and the empirical evidence of international research shows the difficulties in bridging the gap between research and practice [3]. Although norms are difficult to teach, an educational program directed at health-care professionals, social workers, policemen, and politicians could help increase these parties' knowledge of contemporary research, but to really bring about change, multilevel interventions are probably needed [24].

The focus group participants considered the mass media to have an important role to play in building norms among the general public. Evidence for this has been found in South Africa where the media was found to be a powerful tool for creating behavioral change concerning IPV through “edutainment,” in which social issues are systematically integrated into an entertainment format and broadcast on primetime radio or television [24]. However, mass media also carries the risk of evoking undesirable attitudes to IPV by presenting images of stereotypical gender roles. Previous research has shown that news coverage of IPV is limited and has little potential to influence policy or individual behavior [25].

### Reluctance to Take Responsibility for Prevention

A somewhat surprising finding was the lived experience of the professionals that showed how closeness and distance to IPV influenced the way they viewed their involvement in prevention activities. The professionals who frequently encountered IPV in their daily work regarded prevention work as more suitable for organizations other than their own. On the other hand, those who had knowledge about the problem, but no everyday contact with it, acknowledged that they could contribute to prevention, either by themselves or through their organization. Referral of responsibility might be avoided by clarifying who is actually responsible for prevention of IPV. According to Hamilton [26], responsibility refers to liability for sanctions which are based on rules. Attribution of responsibility (saying that it is someone else's problem) is therefore a function of rules and expectations of others. There is an important difference between the legal rules for ascribed responsibility and commonsense notions in this respect. Social roles in society determine in many ways the expectations of responsibility. In order to increase awareness of organizational responsibility, vicarious liability should be enhanced. Furthermore, to acknowledge one's own responsibility, one must clearly understand the expectations of others [27] and be willing to accept these expectations. Comprehensible policies could provide information and help to form an opinion or make a decision.

### Prepared to Take Action

One of the perceived main hindrances for norm building against IPV was shame related to IPV. Therefore, efforts should be made to reduce the stigma, shame, and denial in connection with IPV per se. Talking openly about the problem and encouraging the use of available support services can assist in overcoming the barriers experienced by the victims of IPV [28]. In this study, as in previous studies, men's violence against women was surrounded by silence. However, the focus group discussions showed that both the politicians and the representatives of local trade and industry were prepared to break their silence and contribute to an increased public awareness of IPV. Although initiating such conversations might be difficult, a possible way to do this was identified by one of the politicians who had come to the conclusion that she herself had a responsibility to keep repeating the importance of prevention of IPV in her own speeches and campaigns. This idea is supported by research that identifies key actors, politicians, or other influential authorities as important norm builders [3].

### Strengths and Limitations

To our knowledge, this is the first study that includes different professional groups and decision makers in group conversations about the prevention of IPV. Focus groups were chosen as the method for data collection since conversation in group provides an opportunity for participants to broaden and deepen the topic during the discussion as a result of the interaction. The participants represented a diversity of ideas and experiences which led to dynamic discussions. Occasional statements were ascribed value in the analysis, and the categories were based on variations rather than frequency of statements. In addition, the analysis was carried out first by two researchers with different professional backgrounds and then reviewed and discussed by the other coauthors.

The perceptions and beliefs among professionals and decision makers found in this study are supported to some extent by previous studies, which indicate an acceptable trustworthiness. A weakness worth noting is the selection bias, i.e., the participants were particularly interested in issues concerning IVP and thus knowledgeable in the topic which might have influenced the results. Still, the ideas, opinions, and experiences of the focus group participants may serve as encouragement and create ideas for both practice and further research.

In conclusion, this study showed that professionals and decision makers advocated two different types of prevention. The first was building social structures through political decisions as a way to reduce IPV and to develop social norms against IPV in different public health arenas. The professionals were reluctant to participate in work with IPV while decision makers considered themselves appropriate for the task. This study gave new insights in the prevailing perceptions of professionals and decision makers, which can be an important and useful knowledge in future preventive work and contribute to bridge the gap between research and practice. Future studies are needed to validate the findings in other contexts and to estimate how common they are. IPV is a major public health problem and the need to develop preventive measures is acute given the devastating health and social effects on exposed women, their children, and families.

## Footnotes

1 Author's comment
